# Effect of Isolating the Periosteum With a Resorbable Barrier Membrane on Neoangiogenesis in Guided Bone Regeneration: An Experimental Study

**DOI:** 10.7759/cureus.81069

**Published:** 2025-03-24

**Authors:** Danai Anna Papavasileiou, Minas Leventis, Georgios Agrogiannis, Demos Kalyvas

**Affiliations:** 1 Department of Oral and Maxillofacial Surgery, Dental School, National and Kapodistrian University of Athens, Athens, GRC; 2 1st Department of Pathology, Medical School, National and Kapodistrian University of Athens, Athens, GRC

**Keywords:** angiogenesis, animal models, barrier membranes, collagen fleece, collagen membrane, guided bone regeneration, osteogenesis

## Abstract

Introduction

In guided bone regeneration, the use of bone substitutes protected by resorbable barrier membranes is suggested for the treatment of bone defects. Sprouting of vessels from surrounding local bone and periosteum into such regenerated areas is an important factor for osteogenesis. However, isolating the grafted area from the overlying periosteum with a barrier membrane may affect the revascularization of the site, thus compromising new bone formation.

Aim

The aim of this experimental study was to perform a comparative evaluation of neoangiogenesis in bone defects filled with a bone graft and covered with a resorbable barrier membrane or a collagen fleece.

Method

Eighteen 2.5-3.5 kg weighing New Zealand white rabbits were used. Two circular bicortical bone defects (8 mm in diameter) were created in the calvaria of the animals and subsequently filled with a deproteinized bovine bone mineral ([DBBM]; Bio-Oss®, Geistlich Pharma AG, Wolhusen, Switzerland). One grafted defect was covered with a resorbable collagen membrane (Bio-Guide®, Geistlich Pharma AG) (group A), while the other site was covered by a collagen fleece (Jason® collagen fleece, Botiss Biomaterials GmbH, Zossen, Germany) (group B). The rabbits were divided into three study groups (7, 14, and 28 days), each containing six animals. Specimens were taken, and histological and immunohistological analyses were carried out concerning the number of newly formed vessels.

Results

All specimens showed uneventful bone formation at 28 days. There was a statistically significant difference in the number of blood vessels for the collagen fleece group at all time periods (7, 14, and 28 days).

Conclusions

The results of this study indicate that covering a bone graft with a quickly resorbable biomaterial (collagen fleece) allows for a greater degree of neoangiogenesis compared to a resorbable collagen barrier membrane.

## Introduction

Bone grafting is a routine procedure to manage osseous defects of the alveolar ridge caused by trauma, natural bone loss after tooth extractions, and periodontal disease either around teeth or dental implants.

Guided bone regeneration (GBR) is one of the most widely used and well-documented techniques used on bone defects of the alveolar ridge or around dental implants, concerning the use of barrier membranes along with bone grafts.

The most common biomaterial used in GBR procedures is collagen of animal or human provenance (type I/III). It has proven to have higher properties compared to other resorbable biomaterials. It favors the blood clot formation and acts as a chemotactic for the fibroblasts of the periodontal ligament [1]. Furthermore, research has shown that osteoblasts have better attachment to collagen membranes compared to membranes of different materials, thus favoring bone regeneration [2, 3]. Collagen barrier membranes are absorbable with various rates of degradation, which can be manipulated through processing techniques and may vary from five to nine months [4].

Another form of collagen used in everyday dental/surgical practice is collagen fleece. It is used as a hemostatic, as its structure provides a scaffold for platelets and helps form a stable blood clot. It has a much faster degradation rate compared to a collagen membrane: two to four weeks. However, the literature provides proof that collagen fleeces have been successfully used in GBR, more specifically in sinus lift procedures [5,6].

Bone regeneration in GBR

The formation of new bone in GBR follows the basic principles of osteogenesis during the intramembranous ossification [1]. A prerequisite for the success of bone regeneration procedures and the formation of vital new bone is the vascularization of the grafted area. Angiogenesis plays an integral part in the development and regeneration of osseous tissue [7]. Newly formed blood vessels provide the grafted area with nutrients, oxygen, mesenchymal stem cells, immune system cells, and growth factors. Angiogenesis involves bridging, sprouting, and forming anastomoses between pre-existing blood vessels to form a fully developed and functional vasculature. Neoangiogenesis in adults follows the same mechanisms that are responsible for the formation of the primitive vascular network during fetal development. To measure and quantify angiogenesis, a variable called microvessel density (MVD) was introduced, primarily in oncological studies. It measures the number of blood vessels per square millimeter and is commonly used to assess the prognosis of a tumor or the process of angiogenesis in general [8-10].

Role of periosteum in angiogenesis and osteogenesis

The periosteum plays an important role in bone development and regeneration, and it is inextricably linked with the vascularization of bone and skeletal muscles. It comprises two layers, the outer, fibrous one that gives it its structural stability and an inner, cellular layer that has significant osteogenic potential. This inner layer, also referred to as cambium, is a cellular layer consisting of mesenchymal stem cells, osteoprogenitor cells, osteoblasts, fibroblasts, and endothelial cells. It is responsible for the periosteum's contribution to the development, healing, and regeneration of bone tissue. The vasculature of the periosteum accounts for 70-80% of the blood supply of cortical bone, as the periosteum capillaries are connected to the blood vessels of the underlying cortical bone. Several studies have been dedicated to the association of the periosteum to bone regeneration. The cells of the periosteum have been found to activate the expression of vascular endothelial growth factor (VEGF), which facilitates revascularization, which is a prerequisite for bone regeneration [11-14].

This article was previously presented as a meeting abstract and poster at the 2018 European Association for Osseointegration Annual Congress on October 12, 2018.

## Materials and methods

A total of 18 mature New Zealand white rabbits (mean body weight: 2.5-3.5 kg) were used for this study. Rabbits are a common animal model for testing bone grafting materials and GBR procedures due to their fast skeletal turnover, similarities to human cancellous bone, and well-established critical-size defect models in the calvaria and tibia. They are also easy to handle, cost-effective, and allow for shorter study durations compared to larger animals, making them a practical choice for preclinical research [15].

The study was approved by the Veterinary Directorate of Athens Prefecture, the Committee for the Evaluation of Experimental Protocols of the Laboratory of Experimental Surgery and Surgical Research “N.S. Christeas,” Athens Medical School, Athens, Greece, as well as the Protocols Evaluation Committee and consultation of Institutional Animals Welfare Body of the same institution (IRB number 2662, 30/04/2013). The experimental protocol was submitted to the Veterinary Directorate, General Division of Agricultural Economy & Veterinary Medicine, region of Attica, Greece.

The research protocol was designed following the European Communities Council Directive (2010/63/EU) to apply the 3Rs (Replacement, Reduction, Refinement) in everyday practice [16]. It also used the international guidelines “ARRIVE” (Animal Research: Reporting of in vivo Experimental Guidelines).

The rabbits were housed in individual cages under controlled conditions and given a balanced diet, with free access to water and food. All animals were given seven days to acclimate to the new conditions in the research laboratory. The rabbits were randomly divided into three experimental groups of 7, 14, and 28 days of healing, accordingly, at which point they were going to be sacrificed.

Surgical procedure

**Figure 1:**
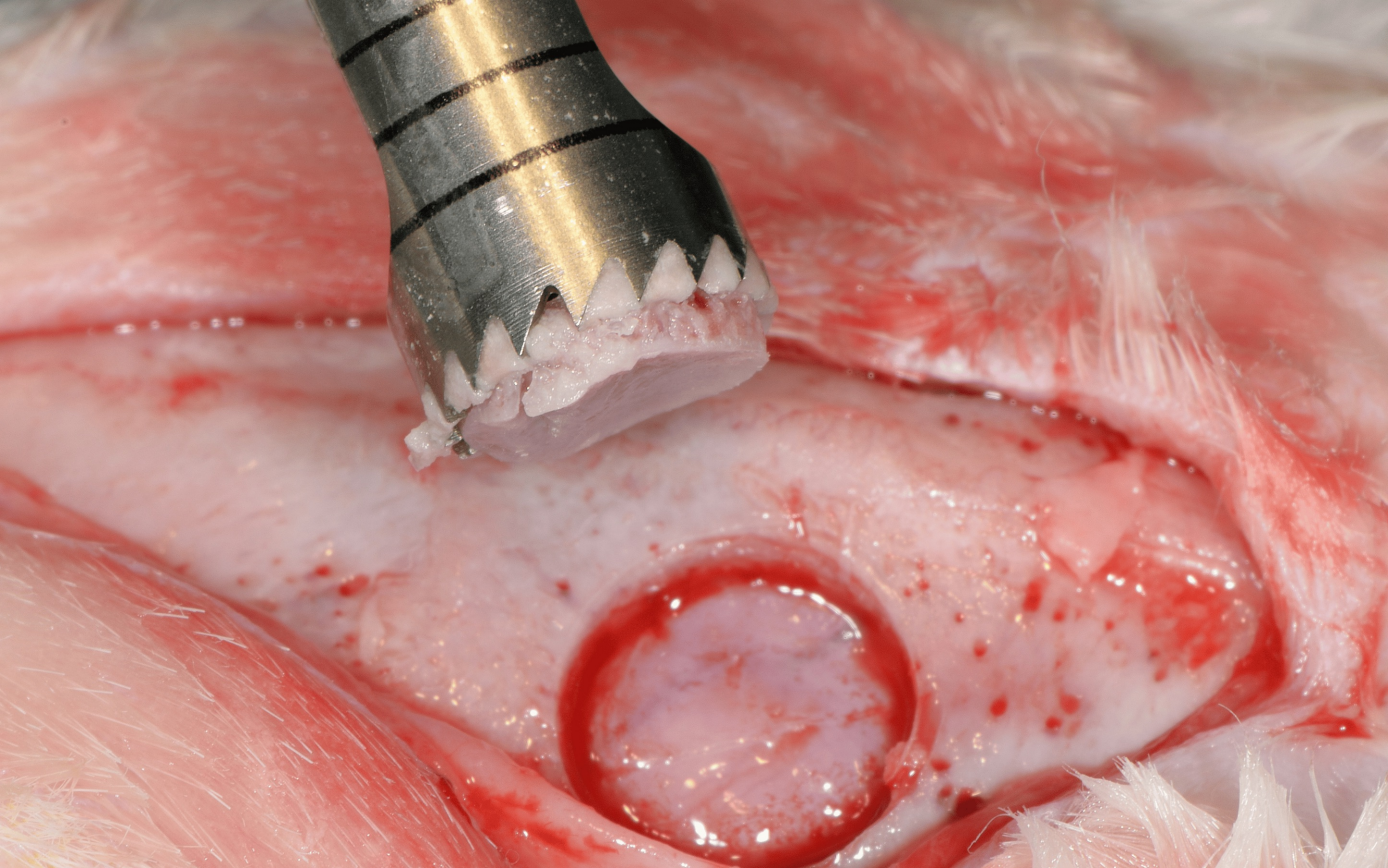
Preparing the circular bone defects with an 8-mm trephine bur.

**Figure 2:**
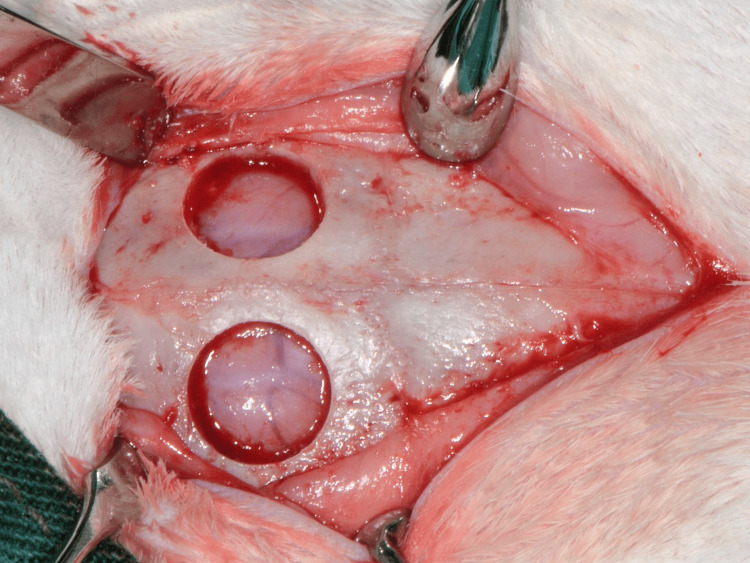
The two bone defects on the parietal bones of the rabbit calvaria. The sagittal suture is visible.

General anesthesia was performed with an intramuscular injection of xylazine (Rompun 20 mg/mL, 5 mg/kg, Bayer Animal Health, Leverkusen, Germany) and ketamine (ketamine hydrochloride, Imalgene 100 mg/mL, 10 mg/kg, Merial, Lyon, France). The designated surgical field was depilated on the calvaria of each rabbit and disinfected with povidone iodine (Betadine Solution, Lavipharm, Athens, Greece). An incision along the sagittal suture was made on the skull of each animal, and the periosteal flaps were reflected to reveal the parietal bones. A trephine surgical bur (8 mm in diameter) (Komet Inc., Lemgo, Germany) under continuous saline irrigation was used to prepare the two circular calvaria bone defects on either side of the sagittal suture (Figures 1, 2). All the defects were prepared without injuring the underlying dura mater.

**Figure 3:**
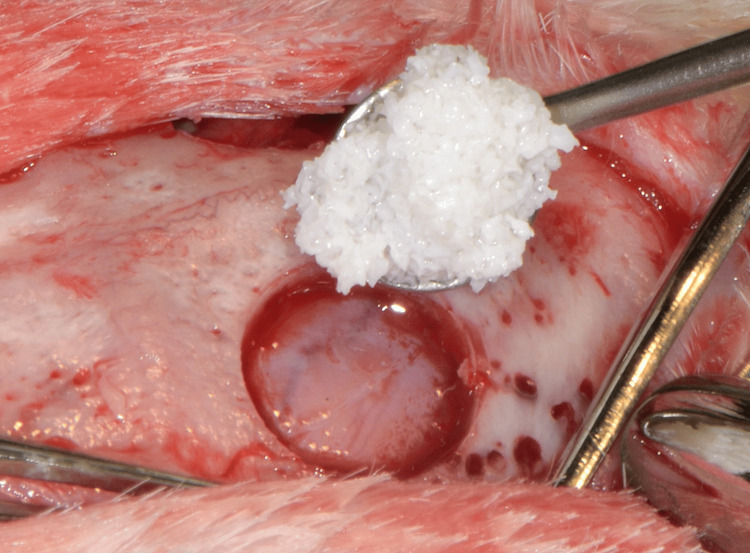
The bone defects are filled with the DBBM graft. DBBM, deproteinized bovine bone mineral

**Figure 4:**
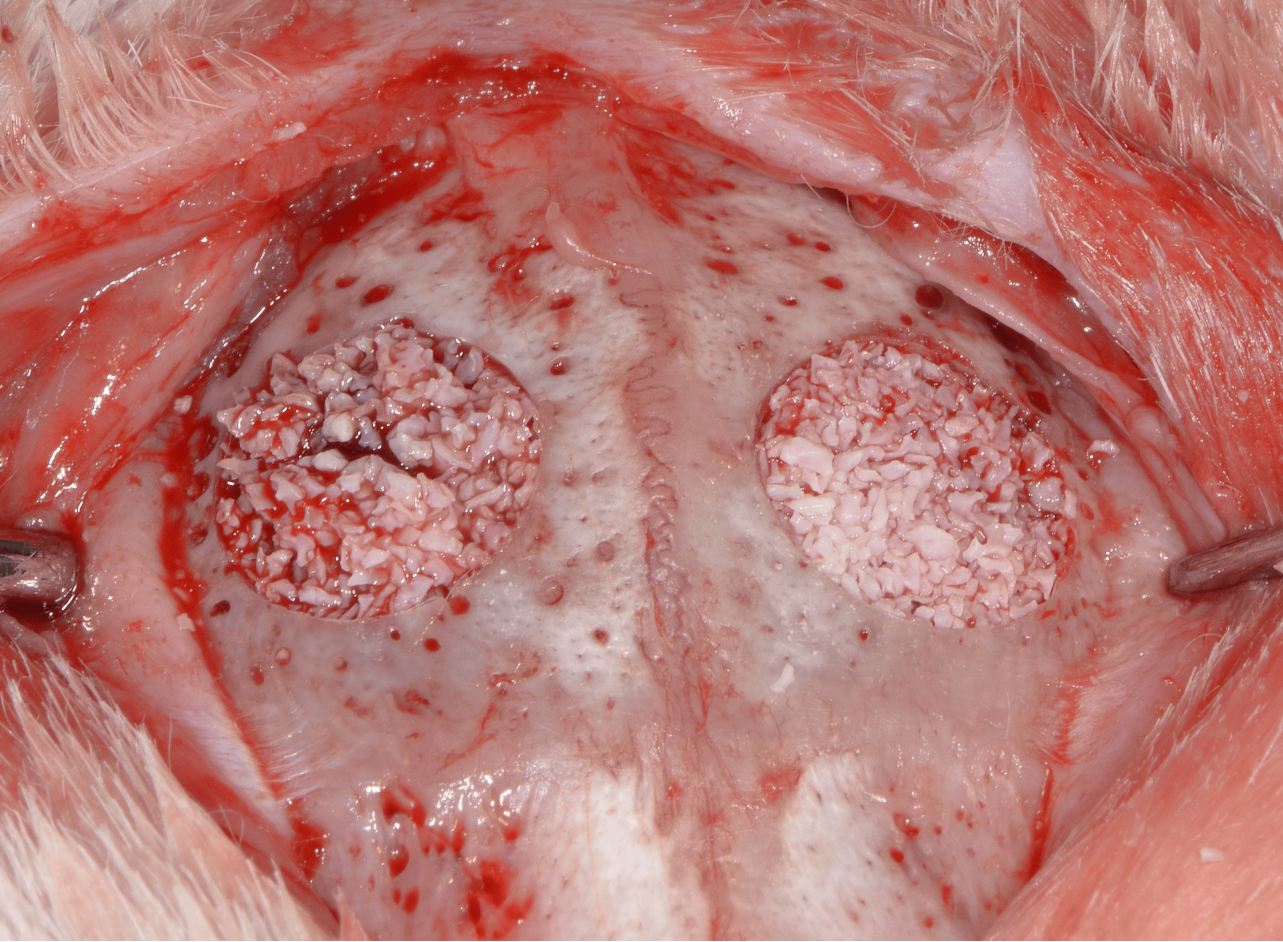
The bone defects are filled with the DBBM graft. DBBM, deproteinized bovine bone mineral

**Figure 5:**
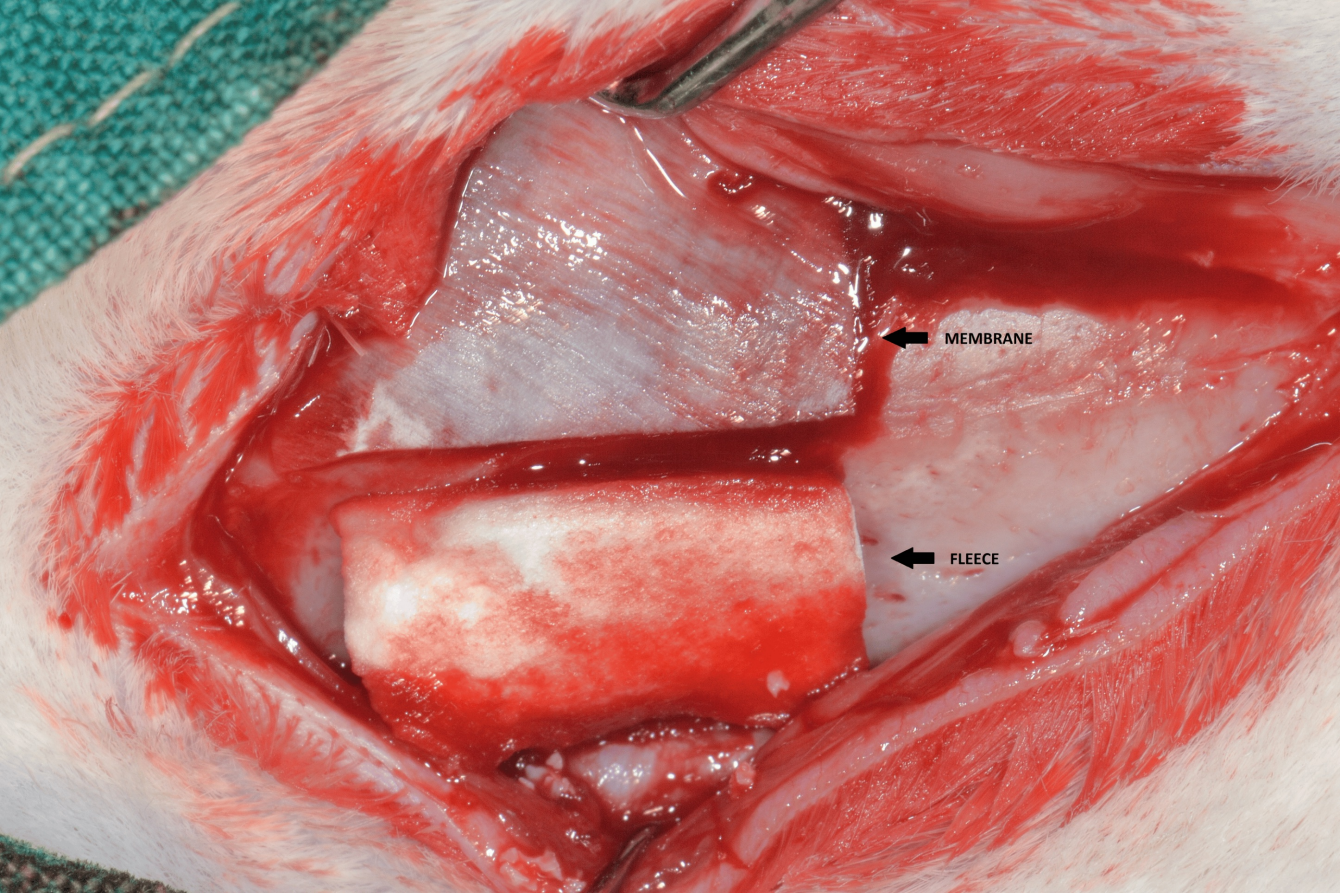
One of the defects is covered with a resorbable collagen barrier membrane and the other one with a collagen fleece.

A demineralized bovine bone mineral (DBBM) was used to fill the defects (Bio-Oss®, Geistlich Pharma AG, Wolhusen, Switzerland) (Figures 3, 4), which were then covered on the left side with a resorbable collagen membrane (Bio-Guide®, Geistlich Pharma AG) (group A). The right side was covered with a collagen fleece (Jason® collagen fleece, Botiss Biomaterials GmbH, Zossen, Germany) (group B) (Figure 5).

Wound closure was performed in layers, with resorbable polyglycolic acid sutures, 4-0 (Coated Vicryl, Ethicon, Johnson & Johnson, Somerville, NJ, USA). Post-operative care for the rabbits included antibiotic treatment daily (30 mg/kg Zinadol, Glaxo Wellcome, Athens, Greece) and analgesic-anti-inflammatory treatment with carprofen (carprofen, Rimadyl, Pfizer, New York, NY, USA) once a day during the course of three days. Two of the rabbits did not recover from the surgical procedure (one from the seven-day study group and one from the 28-day study group). The immediate postoperative condition of the rest of the animals was uneventful, although a few days later two of the rabbits presented with wound suppuration (both belonging to the 14-day study group).

**Figure 6:**
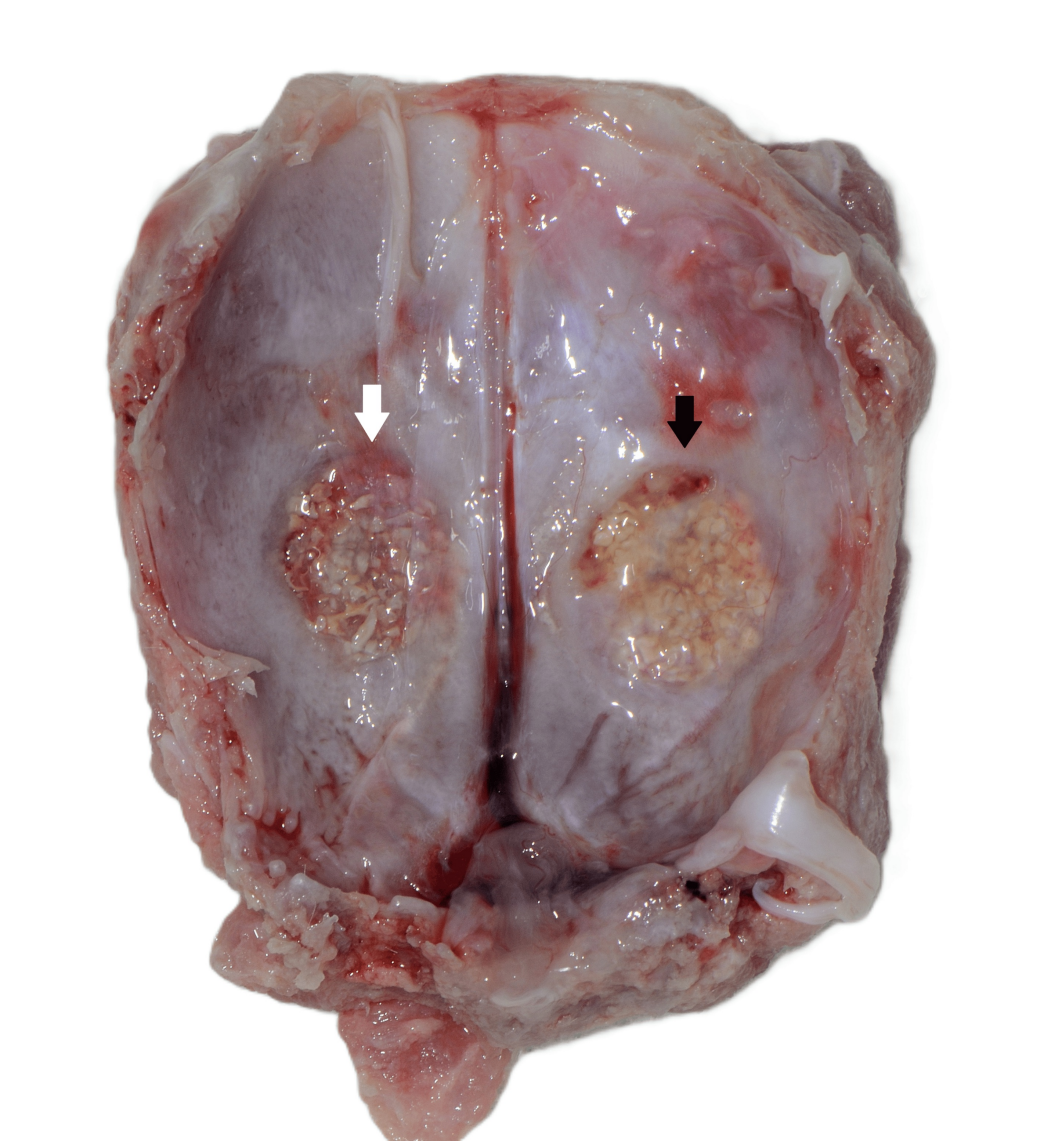
Inner side of the rabbit calvaria specimen 14 days after surgery. The white arrow indicates the collagen membrane side, and the black arrow indicates the collagen fleece side

The euthanasia of the rabbits followed, according to the study group they belonged to, at 7, 14, and 28 days. It was performed under general anesthesia (combination of xylazine and ketamine) with an intravenous administration of sodium thiopental (Pentothal® 100 mg/kg). Subsequently, and under sterile conditions, the calvarium of the rabbits was prepared and a bone block was obtained, containing the sites of the defects created formerly (Figure 6). The retrieved specimens were fixed in 10% neutral buffered formalin solution (formaldehyde solution, Sigma Aldrich, St. Louis, MO, USA). The two bone defects on each calvarium will be hereafter called “group A” (bone defect covered with collagen barrier membrane) and “group B'' (bone defect covered with collagen fleece).

Histological analysis

After fixing the specimens in formalin solution followed the process of decalcification in an EDTA-based solution (MicroDec, Diapath, Martinengo, Italy) for seven days and then they were embedded in paraffin blocks (Tissue Processor, EMBED 503, Kaltek, Padova, Italy).

Six sections were obtained from each bone block: three of them were stained with hematoxylin and eosin stain and subjected to microscopic examination, and the rest were immunostained with a monoclonal antibody against CD31 (Dako, Glostrup, Denmark), which is a marker of vascular endothelial cells. All sections were analyzed by a single pathologist, who was blinded to the treatment groups.

Immunohistochemistry

Immunohistochemistry was applied on 4-μm-thick sections, obtained with a microtome (Microtome RM, Leica, Buffalo Grove, IL, USA). After heating slides at 37°C overnight, slides were deparaffinized in xylene and rehydrated through graded alcohol solutions. Endogenous peroxidase activity was blocked by reaction with 0.3% H2O2 in tris-buffered saline for 30 minutes at room temperature. Antigen retrieval was succeeded by heating the slides in 10-mM citrate buffer (pH 6.0) at 750 W for 15 minutes (two cycles, 5 minutes each) in a microwave oven. After rinsing with tris-buffered saline, normal horse serum was applied for 30 minutes to block nonspecific antibody binding. Subsequently, sections were incubated overnight at 4°C with the primary antibody (anti-CD31, clone M0823, Dako) in a dilution of 1:50. A two-step technique was used (Envision, Dako). For the secondary antibody in this method, antimouse enzyme labelled dextran polymers (DAKO, K4000) were allowed to react for 30 minutes at room temperature. 3,3-diaminobenzidine tetrahydrochloride was used as the chromogenic substrate, and, finally, the slides were slightly counterstained with hematoxylin. A positive internal control for immunostaining was used for each section.

Image analysis

Slides were digitized using a photomicroscope (Nikon Eclipse 80i, Nikon Corp, Tokyo, Japan) with an attached digital camera (Nikon DS-2MW) with a resolution of 1,600x1,200 pixels. The images were then transferred to a computer equipped with appropriate software (Image ProPlus v5.1, Media Cybernetics Inc., Rockville, MD). An evaluation of angiogenesis was then performed using a technique initially established for quantifying tumor angiogenesis by identifying the three most vascular areas (hot spots) with the highest number of microvessel profiles by scanning each section at low power (×20) and then computer analysis of these areas. The digitized images were processed with the aforementioned program, where the newly formed vessels were selected automatically, and their total number and surface area were calculated. The retrieved data were filed in an Excel document.

Statistical analysis

Data were expressed as mean ± standard deviation (S.D.), and the Kolmogorov-Smirnov test was used for normality analysis of the parameters. The comparison of variables between the groups was performed using the paired samples t-test and that between time measurements for each group was performed using the one-way ANOVA model and the Bonferroni test. All tests were two-sided, and statistical significance was set at p<0.05. All analyses were carried out using the SPSS Statistics 17.0 (Statistical Package for the Social Sciences, SPSS Inc., Chicago, IL, USA).

## Results

Overall

Eighteen New Zealand white adult rabbits were used for this study. No complications were noted during the surgical procedure in any of the experimental animals. Two of the animals did not recover from the procedure (one from the 7-day study group and one from the 28-day study group). In the postoperative monitoring period, two of the rabbits presented with wound suppuration (both belonging to the 14-day study group). The rest of the animals had an uneventful postoperative course, with normal wound healing. In all the bone defects, the dura mater and brain tissue were not harmed during the surgical procedure and showed no signs of inflammation. No foreign body reaction was observed in any of the specimens. Finally, in all the bone defects of the 28-day study group (longest healing period), full closure and filling with new bone was macroscopically observed.

Analysis of results

The results are presented hereafter in one collective table (Table 1) and several more detailed ones.

**Table 1:** Collective table presenting the experiment’s results (NUM units: vessels/mm²) p-valuebg, p-value between grafts; SD, standard deviation; SE, standard error

Material	Week 1 (7 days)	Week 2 (14 days)	Week 4 (28 days)	Independent of time	Interaction group*time
	Mean±SD	Mean±SD	Mean±SD	Mean±SE	
Fleece	73.99±12.52	70.41±13.05	61.94±20.46	68.78±3.91	0.540
Membrane	56.08±13.58	41.73±6.26	31.98±9.56a	43.26±2.51	
p-valuebg	0.051	0.009	0.044	<0.0005	-

There was no statistically significant interaction between the two factors (p = 0.540), and thus the MVD variable changes during the observation period in the same way for both types of graft. There was a statistically significant difference in the MVD variable between the two grafts at week 1 (p=0.051), week 2 (p=0.009), week 4 (p=0.044), and independent of time (p<0.0005). Comparison between different time measurements of the MVD variable for each graft separately showed a statistical difference only between weeks 1 and 4 (p=0.006) for membrane graft.

**Figure 7:**
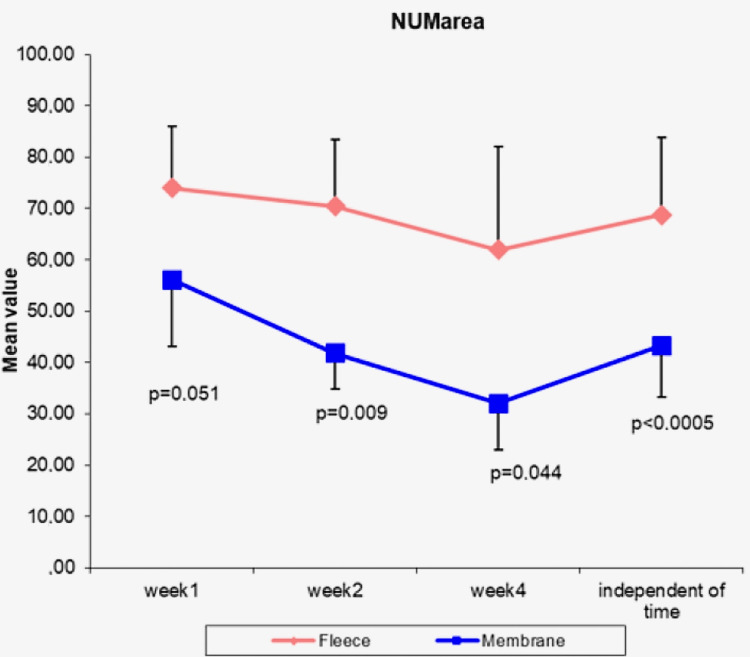
Microvessel density results Comparison between the two materials (collagen membrane and collagen fleece) and the “time” variable during the observation period NUMarea, number of microvessels per mm²

The two-way mixed ANOVA model was used to examine the interaction between the two factors of interest: “time” and “type of covering material” for the MVD variable. There was not statistically significant interaction between the two factors (p = 0.540), and thus the MVD variable changes during the observation period in the same way for both types of material (Figure 7).

Figure 7 shows that there is no statistically significant difference between the two variables (p=0.540), meaning that the MVD variable changes in the same way during the observation period for both types of covering material. This means that the two materials can be studied independently of the “time” variable.

Comparing the two covering materials with the “time” variable

The findings show that there is a statistically significant difference in the MVD variable between the two different material groups (collagen membrane and collagen fleece) independent of “time” (p<0.005) (Table 2).

**Table 2:** Comparison of the two covering materials with the “time” variable (NUM units: vessels/mm²) SE, standard error

Material	Mean	SΕ	p-Value
Fleece	68.78	3.91	<0.0005
Membrane	43.26	2.51	

The comparison of the absolute values for each time point was performed using the paired samples t-test. Comparison between the two covering materials (collagen membrane and collagen fleece) at each individual period of observation (7, 14, and 28 days) was performed using the paired samples t-test.

As shown in Table 3, there is a statistically significant difference between the two covering material groups at the 7-day period (p=0.051), at the 14-day period (p=0.002) and at the 28-day period (p=0.044).

**Table 3:** Comparison between the two covering materials (collagen membrane and collagen fleece) at each individual period of observation (7, 14, and 28 days) (NUM units: vessels/mm²) SD, standard deviation

Material	Mean	SD	p-Value
Week 1			
Fleece	73.99	12.52	0.051
Membrane	56.08	13.58	
Week 2			
Fleece	70.41	13.05	0.009
Membrane	41.73	6.26	
Week 4			
Fleece	61.94	20.46	0.044
Membrane	31.98	9.56	

Finally, the MVD variable for each individual time period was evaluated using the one-way ANOVA model with Bonferroni correction.

Comparative study of the MVD variable for each observation time group

Table 4 shows that there is a statistically significant difference between the individual time periods of observation for the MVD variable for the collagen membrane group (p=0.007). After t-test pairing, a statistically significant difference was observed between the 7-day time period and the 28-day time period (Table 4).

**Table 4:** Comparative analysis of the MVD variable for each study group at 7, 14, and 28 days (NUM units: vessels/mm²)

Material	Week 1		Week 2		Week 4		p-Value
	Mean	SD	Mean	SD	Mean	SD	
Fleece	73.99	12.52	70.41	13.05	61.94	20.46	0.473
Membrane	56.08	13.58	41.73	6.26	31.98	9.56	0.007

Histology

**Figure 8:**
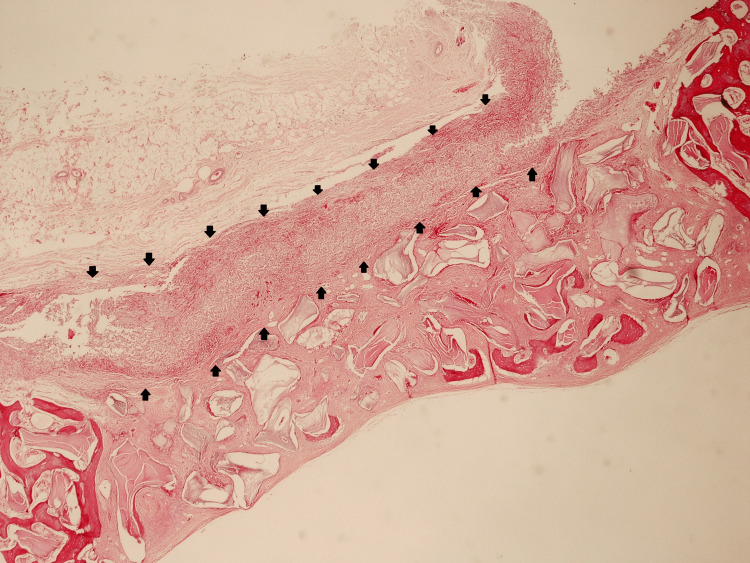
Cross-sectional histological image of group A (collagen membrane + bone graft) 14 days after surgery. Hematoxylin and eosin stain, 100x magnification. Arrows indicate the contour of the collagen membrane.

**Figure 9:**
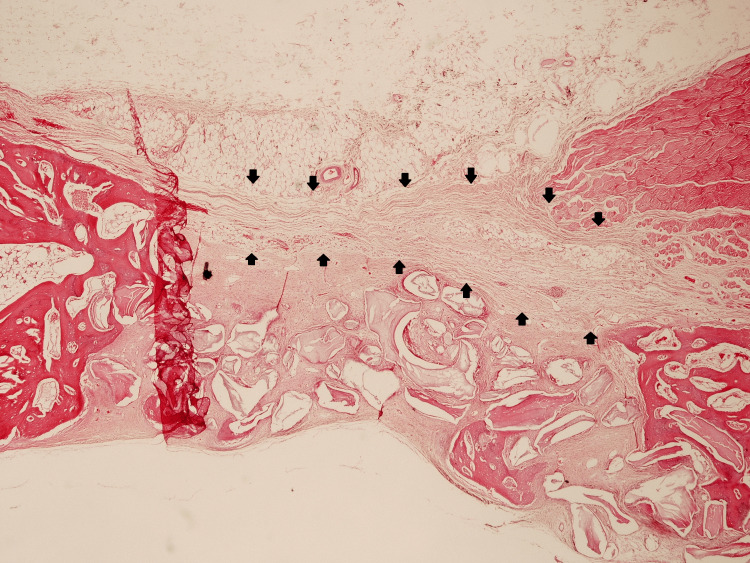
Cross-sectional histological image of group B (collagen fleece + bone graft) 14 days after surgery. Hematoxylin and eosin stain, 100x magnification. Arrows indicate the contour of the collagen fleece.

**Figure 10:**
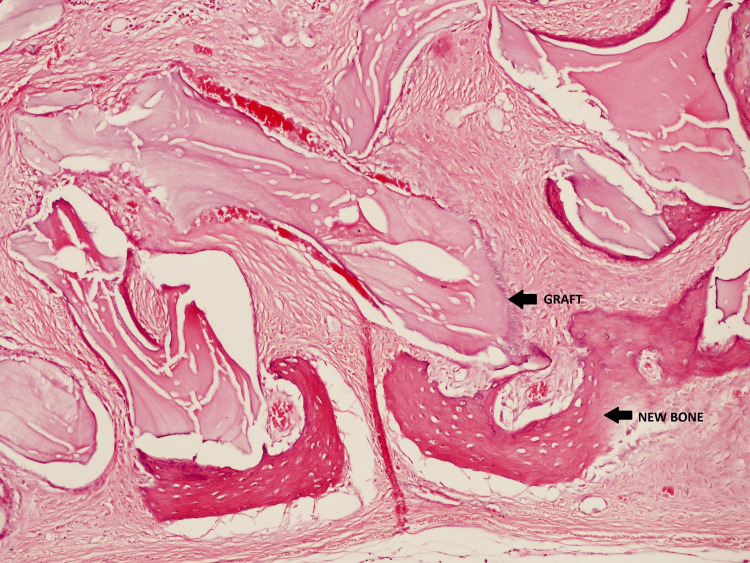
Cross-sectional histological image of the rabbit calvaria 14 days after surgery. Newly formed bone can be seen along with bone graft residue. Hematoxylin and eosin stain, 100x magnification.

The findings from the three study groups (7, 14, and 28 days of observation) are as follows. In the 7-day study group, histological analysis showed highly vascular connective tissue, with no signs of septic inflammation and residual bone graft. In the 14-day study group, gradual replacement of the bone graft by newly formed bone was observed, as well as residual graft in both individual covering material study groups (collagen membrane-collagen fleece) (Figures 8-10). However, the collagen fleece appears to be disintegrating while the membrane maintains its integrity. Finally, in the 28-day study group, there was newly formed bone tissue, residual bovine bone graft, and vascularized connective tissue with no signs of inflammation in all the specimens examined. The new bone is in close contact with the residual graft, encapsulating its particles. No sign of significant inflammation, necrosis, or foreign body reaction was observed in any of the specimens.

**Figure 11:**
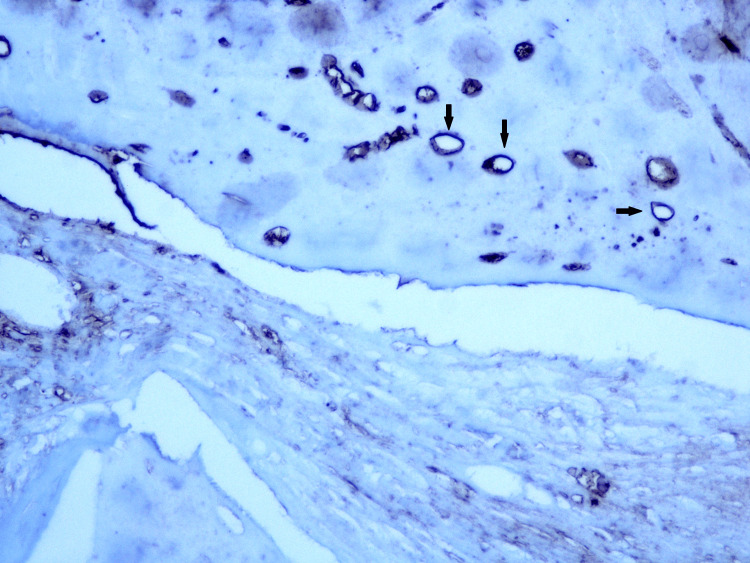
Cross-sectional histological image of the rabbit calvaria 14 days after surgery. Newly formed bone can be seen along with bone graft residue. Hematoxylin and eosin stain, 100x magnification.

The detection of the blood vessels was performed using immunohistochemistry, with a monoclonal antibody against CD31, a marker for vascular endothelial cells (Figure 11).

## Discussion

The purpose of this experimental study was to compare the degree of angiogenesis in osseous defects using a bovine bone graft covered either with an absorbable collagen barrier membrane or with a simple collagen fleece.

The hypothesis is that covering the bovine bone graft filled defect with a collagen fleece will have a positive impact on the number of newly formed blood vessels and the quantity of newly formed bone compared with covering with a collagen barrier membrane.

The two different covering materials compared were the collagen membrane and the collagen fleece, materials of similar provenance but with significantly different properties and degradation rate.

In the present study, the MVD variable was used to quantify neoangiogenesis, measuring the number of newly formed vessels per square millimeter. The MVD variable was used to quantify neoangiogenesis. The correlation between MVD and angiogenesis has been extensively documented in the literature. Galindo-Moreno et al. observed a direct correlation between the number of vessels (MVD) and the quantity of newly formed bone [17]. Yao et al., on the other hand, documented an association between the number of blood vessels per square millimeter (MVD) in new bone tissue and the rate of new bone formation [18]. Artese et al. came to a similar conclusion, corroborating the statement that the success of bone regeneration is directly proportional to the degree of vascularization. In this light, osteogenesis is affected by the angiogenic potentials of the biomaterials used in each GBR procedure [19].

Boëck-Neto et al. in their study on VEGF expression and MVD in GBR procedures of the sinus using various bone grafts underlined the relation between angiogenesis and osteogenesis [20].

In the present experimental study, increased MVD values in all three observation periods were recorded for the collagen fleece group. This indicates a greater degree of neoangiogenesis in the collagen fleece group compared to the collagen membrane study group.

Collagen membranes are the most widely used absorbable barrier membranes. However, their effect on angiogenesis remains unclear until now. Many studies have proven that collagen has higher properties compared to other resorbable biomaterials. Namely, it favors blood clot formation and acts as a chemotactic for the fibroblasts of the periodontal ligament [2,3]. Nevertheless, researchers have reported the negative impact of collagen on angiogenesis. Gunda et al. confirm the existence of a “non-collagenous domain” (NC1) in collagen structure that negatively affects angiogenesis [21]. In another study, the enzyme prolyl hydroxylase enzyme (PHD) acts as an inhibitor of HIF-1a, a transcription factor vital to the signaling path of angiogenesis. In an experimental study on femur fractures in a rat model, inhibition of PHD activity enhanced angiogenesis, thereby promoting osteogenesis. The processing techniques used to enhance the structural integrity of the collagen membranes (e.g., “cross-linking” with ultraviolet radiation, glutaraldehyde, and hexamethylene diisocyanate) have been reported to negatively impact bone healing [2], while this is contested by other authors [22].

All the aforementioned data make it clear that there are aspects of the effects of collagen membranes on angiogenesis and osteogenesis that have yet to be clarified. In this study, a statistically significant difference in the MVD variable was reported (p<0.05) in all the observation periods (7, 14, and 28 days), which demonstrated a higher number of newly formed vessels for the collagen fleece group. Isolation of the periosteum with slowly degrading (or non-absorbable) biomaterials has been found to impact angiogenesis and osteogenesis. Hopper et al. studied the effects of isolating the periosteum on the healing of calvarial onlay bone grafts and reported a statistically significant decrease in the number of new blood vessels, the rate of new bone formation, and the volume of new bone that was measured at 10 weeks postoperatively [23].

The importance of the periosteum as a natural barrier that effectively protects the grafted area from epithelial cell infiltration has been widely studied in the literature. When the vascularized periosteum remains intact and closely contacts the grafted bone defect, it supplies blood, nutrients, and osteoprogenitor cells, promoting earlier neoangiogenesis and osteogenesis [24]. Elsahat et al. investigated the effect of the periosteum as a barrier membrane compared to covering a grafted area with a non-absorbable membrane and found that the areas where the periosteum was used had a higher bone density, while no statistically significant difference in new bone volume was reported between the two study groups [25]. Furthermore, periosteum acts as an enhancer of the osteogenic process, as it provides progenitor cells, osteoblasts, and fibroblasts to the grafted area [13].

The aforementioned points coincide with the findings of this experimental study, where a statistically significant difference in the MVD variable is reported between the 7-day study group and the 28-day study group. This reinforces the hypothesis that these results have a greater importance in the first stages of bone regeneration. At seven days postoperatively, the collagen fleece is already in an advanced state of disintegration compared to the collagen membrane, which maintains its structural integrity, and the grafted defect is in close contact with the periosteum. This allows for a better and earlier development of the vasculature, which could lead to an earlier and enhanced bone regeneration.

Until today, there is limited evidence to support the effectiveness of barrier membranes in the treatment of bone defects [26]. Several studies have been conducted to investigate the use of intact periosteum for bone defect coverage compared to various membranes. Yang et al. compared the degree of bone resorption in autologous onlay bone grafts in a rabbit model when covered by a collagen membrane, by an intact periosteum, or not covered at all. Their results showed no statistically significant difference between the collagen membrane and periosteum group [27]. This leads to the conclusion that the periosteum is just as effective in its use as a barrier for unwanted cell populations as collagen membranes. It favors graft vascularization and its integration in the recipient area and limits the osteoclastic activity that could hinder the bone regeneration process. De Marco et al. in their study of revascularization of autologous bone grafts in a rat model reported that when in contact with intact periosteum, graft revascularization originated from both the bony bed and the surrounding connective tissue and occurred at an earlier stage of the healing process [28]. Ma et al. investigated the role of the periosteum and collagen membrane on bone regeneration of dehiscence-type defects model in beagle dogs. Twenty-four defects were surgically created and subsequently divided into three groups: untreated defects, defects treated with DBBM and a collagen membrane, and defects treated with DBBM and periosteal coverage. Micro-CT, histological, histomorphometrical, and immunohistochemical analyses revealed that the periosteum-covered group showed superior vertical bone gain, greater new bone formation, and enhanced mineralization, indicating that periosteal coverage offers significant advantages over collagen membranes for bone regeneration [29]. However, in another animal study, Kim et al. reported better results when DBBM was covered by a collagen membrane. The mandibular premolars were extracted unilaterally and three ridge defects were created in six mongrel dogs. Each defect site was randomly assigned to one of the following treatment groups: untreated defects, defects treated with DBBM alone, and defects treated with DBBM covered by collagen membrane. Dental computed tomography scans performed at 8 and 16 weeks postoperatively showed significantly higher bone density and more successful bone regeneration in the DBBM + collagen membrane group compared to the group with untreated defects and the group where only DBBM was used [30].

This study has certain limitations. The bone defects in the animal model were created in a sterile environment, which does not fully replicate the more complex and challenging conditions of the oral cavity. While the model provides a useful analogy to human applications, further research is needed to assess the effectiveness of these materials and methods in the clinical settings where they are intended for use.

## Conclusions

This experimental study highlights the significant impact of different collagen-based covering materials on angiogenesis within surgically prepared standardized osseous defects. The findings demonstrate that the use of a rapidly disintegrating biomaterial (collagen fleece) allows for a greater degree of neoangiogenesis compared to the use of an absorbable collagen barrier membrane at all observation periods (7, 14, and 28 days). This suggests that the faster degradation of the collagen fleece allows for earlier vascularization, which could enhance the initial stages of bone regeneration. The study also reinforces the crucial role of the periosteum in bone healing, as its direct contact with the grafted site may promote early neoangiogenesis and osteogenesis. These results align with existing literature, emphasizing the importance of vascularization in successful bone regeneration.

However, given the controlled conditions of this standardized animal model, further research is necessary to validate these findings in everyday clinical settings, where additional biological and mechanical challenges exist. Future preclinical and clinical studies should investigate the long-term effects of collagen-based barrier membranes on angiogenesis and assess the impact on the quantity and quality of the regenerated bone to optimize GBR strategies in clinical practice.
